# Missed diagnoses in African Americans with obsessive-compulsive disorder: the structured clinical interview for DSM-IV Axis I disorders (SCID-I)

**DOI:** 10.1186/s12888-017-1422-z

**Published:** 2017-07-17

**Authors:** Gregory S. Chasson, Monnica T. Williams, Darlene M. Davis, Jessica Y. Combs

**Affiliations:** 1 Department of Psychology, Illinois Institute of Technology, 3105 S. Dearborn, Chicago, IL 60616 USA; 2 Department of Psychological Sciences, University of Connecticut, 406 Babbidge Road, Unit 1020, Storrs, CT 06269-1020 USA; 3 Center for Mental Health Disparities, Department of Psychological & Brain Sciences, University of Louisville, 2301 South Third Street, Louisville, KY 40292 USA; 4 Department of Psychology, Spalding University, Louisville, KY USA

**Keywords:** Obsessive-compulsive disorder, African Americans: Assessment, Race, Structured interviews

## Abstract

**Background:**

Research on the utility of structured interviews in assessing OCD is scarce, and even more so, in its use for OCD in African Americans. The purpose of this study was to examine the utility of the Structured Clinical Interview for DSM-IV Axis I Disorders (SCID-I) in detecting OCD in African Americans when used by well-trained, culturally competent clinicians.

**Methods:**

Seventy-four African American adults with OCD were assessed with the SCID-I and additional measures of OCD.

**Results:**

Results revealed the poor diagnostic utility of the SCID OCD section (SCID-OCD), with 66.2% (*N* = 49) correctly identified and 33.8% (*N* = 25) incorrectly diagnosed. Participants receiving the correct diagnosis were more likely to endorse compulsive behaviors, specifically ordering compulsions, and experience greater symptom severity.

**Conclusion:**

The lack of sensitivity for identification of OCD is discussed as the SCID-OCD seems to often miss a true diagnosis of OCD in African Americans.

## Background

Obsessive-Compulsive Disorder (OCD) is a highly distressing, often debilitating psychological disorder and a major cause of disability worldwide [1]. The total economic cost of OCD is estimated at over eight million dollars, accounting for 18% of the total costs of all anxiety-related disorders [2]. As an important public health challenge, more research is needed to improve the understanding and treatment of OCD, and foundational to this understanding is improving assessment of the disorder, especially in underrepresented minorities.

An estimated 1.6% of the population in the United States suffers from OCD [3, 4]. African Americans account for 13.6% of the population in the United States and have equivalent rates of OCD compared to the general population [5–7], but they are generally underrepresented and underserved in OCD treatment clinics and research studies [8, 9]. Research has shown that several measures assessing OCD lack validity in African American samples, contributing to their failure to properly identify OCD in minority populations and having the propensity to either over- or under-diagnosis this population with OCD [10, 11]. As such, more work is needed to validate clinical measures used to assess OCD symptomology in African American samples, as accurate assessment of the disorder is critical for providing treatment in this underserved segment of the population and to reduce this public health challenge.

### Assessment of obsessive-compulsive disorder

The *Structured Clinical Interview for DSM-IV Axis I Disorders* (SCID-I) [12, 13] is one of the most widely used structured interviews and assesses for the presence of Axis I Disorders, including OCD. Researchers using the SCID-I to assess anxiety and related disorders have found it to be accurate in its detection of disorders, yet also noted its accuracy as largely dependent upon the administrator’s experience for assessing OCD [14, 15]. Authors of the SCID-I recently released the *Structured Clinical Interview for DSM-5 Disorders* (SCID-5) [16], but accessibility is increasing and training is still under development. In addition, the SCID-5 requires more financial investment for use, especially in research, as the cost use is more expensive than for the SCID-I. This cost differential may be important for cash-strapped community clinics, which is often the primary site for mental health services for underrepresented groups. Such clinics drawn to evidence-based assessment may prefer a low cost alternative to the SCID-5. Such a decision is defensible given that the diagnostic criteria for OCD from DSM-IV to DSM-5 was modest, suggesting that OCD diagnoses from the SCID-I could be relevant in a practice setting. As such, the utility of the SCID-IV as a diagnostic tool remains relevant in both clinical and research settings.

The literature examining the accuracy of diagnosing OCD using structured interviews has yielded inconsistent findings explained by interviewer training and level of expertise with OCD [17]. As one example of the importance of the interviewer’s level of OCD expertise, in the National Comorbidity Survey Replication (NCS-R) epidemiological study, respondents tended to misidentify everyday sources of stress and worry as obsessions (for example, worries about finance or relationships might have been incorrectly classified as OCD), resulting in over reporting that lead to over diagnosis. Additionally, both respondents and lay interviewers (trained non-clinicians) overestimated the degree of impairment or distress resulting from OCD symptoms. This issue of accurately diagnosing such a complicated disorder has been previously documented [18–20]. Given the inconsistent results regarding the diagnostic accuracy of the SCID-I, more work is required to determine if OCD can be accurately detected using the SCID-I when administered by trained and experienced professionals.

### Assessment of OCD in African Americans

Research has shown that many measures of OCD do not function as intended in African Americans, (e.g., the Padua Inventories [10] and the Maudsley Obsessional Compulsive Inventory [21]), which makes measures of OCD suspect for this group [11, 22]. Therefore, it is important that all measures of OCD be demonstrated reliable and valid for assessing symptoms of OCD in African Americans [23]. Currently there are no published articles about the utility of the SCID-OCD for African Americans. Studies using the SCID-I to diagnose OCD have rarely reported demographic data or presented correlates or analyses using the race and ethnicity of the research participants [15, 17, 24]. Similarly, other studies utilizing the SCID-I only referenced the race of the participants in broad terms, stating that the majority of the participants were White without identifying the other racial groups in the study [14, 25].

While the SCID-I has not been validated in minority groups, one gold standard measure of OCD, the Yale-Brown Obsessive-Compulsive Scale (Y-BOCS) [26], has been validated and exhibited good psychometric properties in the assessment of OCD in African Americans [27]. Although the Y-BOCS is accurate at providing information about symptom dimensions and severity, it is not intended as a diagnostic tool. There is ultimately very limited research on diagnostic tools for OCD in African American samples; therefore, this study proposes to examine the psychometric properties of the SCID-OCD in a clinical sample of African Americans with OCD. We attempt to determine if the SCID-OCD accurately detects OCD in African Americans when present when assessed by interviewers who are well trained and culturally competent in working with this population.

## Methods

### Participants

Eighty-three adults were recruited for the study of African Americans with OCD at the University of Pennsylvania School of Medicine at the Center for the Treatment and Study of Anxiety (CTSA), an internationally recognized expert specialty clinic for OCD. Recruitment occurred in 2009–2010, over a 9.5-month span [28]. Participants were excluded if they did not have OCD or had an intellectual impairment that made them unable to participate in the assessment process. Seventy-four were determined to have a current diagnosis of OCD or meet criteria for subclinical symptoms that had at one time met criteria for an OCD diagnosis. Nine participants did not meet criteria for OCD and were excluded from analyses.

Of the remaining 74, 42 were female (56.8%) and ranged in age from 19 to 61, with an average age of 41.4 years (*SD =* 12.3). The average household income was $20–39,999 annually. On average, the 49 participants diagnosed correctly (*M* = 39.4, *SD* = 12.08) using the SCID alone were younger than those undiagnosed by the SCID (*M* = 45.1, *S*D = 12.14). Female participants made up 57.1% of the group diagnosed correctly and 56.0% of the group undiagnosed by the SCID. See Table 1 for details. The diagnosis of OCD for each participant was determined using a best-estimate procedure (described in the Procedures section) based on data collected from all the assessment instruments described below. More details about the sample and recruitment process are available elsewhere [28].

**Table 1:** Demographics by Diagnosed versus Misdiagnosed Status

Characteristics	Diagnosed		Misdiagnosed		Total	
	*N*	Percent	*N*	Percent	*N*	Percent
Marital Status						
Divorced, Separated, Widowed	9	18.3%	8	32.0%	17/74	23.0%
Married	12	24.5%	2	8.0%	14/74	18.9%
Single	28	60.0%	15	57.1%	43/74	58.1%
Education						
7–12 Years	6	12.2%	3	12.0%	9/74	12.2%
HS Diploma/ GED	9	18.4%	4	16.0%	13/74	17.6%
Part College/ 2-Year College	19	38.8%	15	60.0%	34/74	45.9%
4-Year College	6	12.2%	3	12.0%	9/74	12.2%
Part or Completed Grad/ Professional School	9	18.4%	0	0.0%	9/74	12.2%
Income						
< $9000	10	23.8%	8	33.3%	18/66	27.3%
$10–39,000	18	42.9%	12	50.0%	30/66	45.5%
$40–79,000	9	21.4%	1	4.2%	10/66	15.2%
$80- > $100,000	5	11.9%	3	12.5%	8/66	12.1%
Comorbidity						
Any Current Anxiety or Trauma-Related Disorder	21	43.8%	9	39.1%	30/71	42.3%
Any Current Mood Disorder	27	58.7%	9	39.1%	36/69	52.2%
Any Current Substance Use Disorder	3	6.1%	1	4.0%	4/70	5.7%
Treatment						
Individual: CBT	6	14.0%	3	17.6%	9/60	15.0%
Hospital/ Inpatient	0	0.0%	2	14.3%	2/54	3.7%
Medication: Primary Care Doctor, Internist, Psychiatrist	6	15.0%	4	28.6%	10/54	11.1%

### Measures

#### Structured clinical interview for DSM-IV Axis I diagnosis (SCID-I)

The entire SCID-I [13] was administered by specially trained master and doctoral-level community clinicians and doctoral-level expert OCD interviewers to assess for major Axis I diagnoses. The interview covered the major mental disorders that include anxiety, substance use, depression, and psychotic disorders.

The SCID-I has a screening form consisting of 24 items assessing symptomology for various Axis I disorders. The screening form prompts the interviewer to “Go To” the specific diagnostic section based on patient responses. For example, if patients select “yes” to either questions 8 (obsessions) and/or 9 (compulsions), the interviewer proceeds directly to the SCID-OCD section to assess for obsessive-compulsive symptoms. However, if the screening form is not used, the interviewer administers every question in each diagnostic section for all disorders assessed by the SCID-I.

In the SCID-OCD, three items assess for the presence of obsessions and two items assess for the presence of compulsions. The responses include *?* (inadequate information), *1* (symptom is absent or is false), *2* (symptom is subthreshold) and *3* (symptom passes the clinical threshold or is true). The SCID-OCD Obsessions section begins with an inquiry, “You’ve said that you have had thoughts that didn’t make sense and kept coming back to you even when you tried not to have them…” If unclear, respondents are provided with examples, “thoughts like hurting someone, even though you really don’t want to or being contaminated by germs or dirt?” If respondents endorse the presence of obsessions, follow up questions seek to gather more descriptive information about obsessive thoughts, assess the level of intrusion and rule out the presence of psychotic symptoms.

The SCID-OCD Compulsions section is similar to the obsession section. If the screener was endorsed, a prompt is administered, “You’ve said that there were things that you had to do over and over again and couldn’t resist doing, like washing your hands again and again, counting up to a certain number or checking something several times to make sure that you had done right…” When the screener has not been used as part of the interview, the client is read the prompt, “Was there ever anything that you had to do over and over again and couldn’t resist doing, like washing your hands again and again, counting up to a certain number, or checking something several times to make sure that you’d done it right?”

If any item assessing symptom presence is scored as *3*, the patient is prompted to describe the nature of the obsessions/compulsions. Follow-up questions include age of onset, level of distress and whether or not the patient takes medication. Upon completion of both sections, the interviewer is prompted to diagnose and indicate severity.

#### Yale-Brown obsessive-compulsive scale (Y-BOCS)

The Y-BOCS Checklist and Severity Scale [26] were administered to participants by interviewers to assess OCD symptoms. The severity scale rates the time occupied by obsessions and compulsions, how much they interfere with functioning, how much distress they cause, attempts to resist, and level of control, with the first five items addressing obsessions and the remainder focused on compulsions. Items are rated on a 5-point scale ranging from 0 (no symptoms) to 4 (severe symptoms). The Y-BOCS severity scale shows good reliability (α = .88–.91) and validity in European American samples [29, 30]. Scores above 16 may be considered in the clinical range, and the mean for OCD patients is 21.9 (SD = 8). The Y-BOCS accurately assesses OCD symptom severity in African Americans (α = 0.83) [27].

#### Brown assessment of beliefs scale (BABS)

The Brown Assessment of Beliefs Scale [31] is a seven-item, semi-structured interview that assesses the degree of conviction and insight patients have about the beliefs underlying their obsessional thinking. The BABS exhibited good reliability in this sample (α = 0.83).

#### Obsessive beliefs questionnaire-brief version (OBQ-44)

The Obsessive Belief Questionnaire – Brief Version is a 44-item self-report measure that assesses cognitive beliefs in OCD [32]^.^ The measure was revised using 44 items taken from the OBQ-87 to establish three subscales [33]. Items were scored from 1 to 7 and summed for a total score. The OBQ-44 consists of the following subscales: (1) responsibility and threat estimation, (2) perfectionism and intolerance for uncertainty, and (3) importance and control of thoughts. It has very good reliability in African Americans (α = 0.94–0.96) [34].

#### Obsessive-compulsive inventory- revised (OCI-R)

The Obsessive-Compulsive Inventory-Revised is an 18-item self-report measure that yields a profile of distress over the past month for each symptom area in the six subscales: washing, checking, ordering, obsessing, hoarding, and neutralizing [35]. Items on the OCI-R were scored from 0 to 4. The OCI-R adequately assesses distress and symptoms dimensions in African Americans (α = 0.92) [34], albeit with higher cut-off scores than in European Americans, and was included to examine symptom dimensions and severity.

#### Beck depression inventory-II (BDI-II)

The Beck Depression Inventory-II is a widely used 21-item self-report measure of depressive symptoms [36]. The BDI-II is an adequate measure of depressive symptoms in African Americans [37] and exhibited good reliability in the current sample (α = 0.93).

#### Global assessment of functioning (GAF)

The Global Assessment of Functioning is a scale used to evaluate severity of illness and overall psychosocial functioning, taking into account psychological, occupational, and social functioning [38, 39]. The scale ranges from 0 to 100, measuring psychiatric impairment with 0 representing extreme impairment and 100 representing exceptional functioning. GAF ratings were determined by interviewers based on the information gathered during clinical interviews.

#### Clinical global impression (CGI)

The Clinical Global Impression is a scale used to evaluate overall symptom severity from OCD only. Severity of participant OCD symptoms was reported on a Likert scale from 0 to 7, with the higher score representing greater the symptom severity [38, 39]. CGI scores were determined by the evaluators and based on information gathered through clinical interview.

### Procedures

Participants for this study were recruited through newspaper ads, radio, public transportation, Internet, and flyers. This study was conducted in compliance with the university Institutional Review Board. Trained research assistants screened prospective participants by phone, and those who reported significant distress or impairment due to their symptoms of OCD were invited to participate in the study. Research assistants had extensive training and advanced knowledge of OCD, thus they were highly effective at distinguishing OCD from other psychopathologies, making a correct diagnosis in over 90% of cases assessed. Once screened, study personnel obtained written informed consent and comprehensive demographic information, and provided supports while participants completed self-report measures. These measures were used to collect data on OCD symptom dimensions, anxiety symptoms, cognitive belief patterns, and depressive symptoms.

Study evaluators were master’s or doctoral-level clinicians. African American clients generally prefer to be ethnically matched to their therapist [40], thus whenever possible African American evaluators were used to create a familiar environment where participants could feel comfortable [41, 42]. Because some research has indicated that African American participants may be uncomfortable in a university-setting, the primary evaluators were therapists who were practicing in the local African American community and not otherwise employed by the university at which the study was conducted. All evaluators received extensive training before assessing study participants, observing a minimum of two complete evaluations conducted by the principal investigator (which included an unstructured clinical interview, the SCID-I, and Y-BOCS) and conducting two study evaluations with the principal investigator (PI) present before being considered trained for the purpose of this study. Evaluators attended reliability meetings regularly and a training workshop led by an expert senior researcher (CTSA director) about the assessment and treatment of OCD. Evaluators met regularly with the PI for study supervision, with a focus on diagnostic and cultural issues [30].

Participants met with interviewers who administered a comprehensive psychiatric diagnostic interview that included an unstructured clinical interview, the SCID-I, Y-BOCS, CGI, BABS, and GAF, among other interview instruments as part of a larger study (for more details, see [30]; only instruments pertinent to the diagnostic process are described in the current study). When administering the SCID-I, the screener was completed prior to the interview, but interviewers were told to administer the entire OCD section of the SCID-I regardless of screening form question responses. Additionally, all interviews were video recorded and reviewed by the PI. Diagnoses were established using a best-estimate procedure [43] in which study staff synthesized clinical data from all sources (e.g., interviews and self-report instruments; see [30] for a full description of the procedure). Inter-rater agreement for an OCD diagnosis was obtained for one-third of the sample using an expert OCD clinician, deemed adequate (agreement in 17 out of 18 cases), and is discussed in a previous report [30]. In situations in which the diagnostic raters disagreed, the PI made a final determination using the best-estimate procedure.

Participants meeting criteria for OCD were offered referrals for treatment. Participants were paid $100 for participation in the assessment and $10 for transportation costs. All participants received a follow-up phone call after a minimum of 4 weeks to determine if they were successful in obtaining effective treatment for their OCD [30].

### Data analysis

Multiple pairwise comparisons were conducted to compare differences between participants with OCD who were correctly diagnosed versus incorrectly diagnosed. With categorical dependent variables (i.e., response on the SCID-I screening form and SCID-OCD), multiple chi-square tests and Fisher’s Exact tests of independence were performed. With continuous dependent variables, such as Y-BOCS and OCI-R, multiple independent sample *t*-test were performed to examine group differences. Alpha was not adjusted to accommodate multiple pairwise comparisons because doing so would have adversely impacted statistical power. With a current sample size of 74, the study was sufficiently powered to detect a medium effect size (*W* = .315, alpha = .05, beta = .80) or larger. Splicing alpha would have over-conservatively masked effects due to low statistical power. Effect sizes were calculated to measure the strength of group differences on measures and subscale total scores.

## Results

Descriptive statistics revealed poor diagnostic ability of the SCID-OCD, as shown in Table 2. Of the 74 participants with clinically elevated Y-BOCS scores (*M* = 23.47, *SD* = 14.46), 49 were correctly diagnosed, meeting criteria for OCD using the SCID-OCD and 25 participants were not diagnosed with OCD using the SCID-OCD despite those participants presenting with bona fide OCD.

**Table 2:** Item Response Differences between African Americans Correctly and Incorrectly Diagnosed for OCD using the SCID-I

SCID-OCD Section	Diagnosed Responses			Misdiagnosed Responses		
SCID Screener						
Screen #8- Obsessions	No: 34.7%		Yes: 65.3%	No: 16.0%		Yes: 84.0%
Screen #9- Compulsions*	No: 4.1%		Yes: 95.9%	No: 20.0%		Yes: 80.0%
SCID-OCD Section						
F84a Screen #8 Prompts*	No: 26.5%		Yes: 65.3%	No: 8.0%		Yes: 88.0%
F85 Thoughts and Marked Distress	1: 4.1%	2: 6.1%	3:77.6%	1: 8.0%		3: 64.0%
F86 Thoughts not Excessive Worries	1: 8.2%	2: 2.0%	3:67.3%	1: 12.0%	2: 8.0%	3: 52.0%
F87 Attempt to Ignore/Suppress	1: 10.2%	2: 4.1%	3:63.3%	1: 20%	2: 4.0%	3: 48.0%
F88 Thoughts not Own	1: 6.1%	2: 2.0%	3:67.3%	1: 8%		3: 64%
F88a Screen #9 Prompts	No: 4.1%		Yes: 89.8%	No: 12.0%		Yes: 84.0%
F89 Repetitive Behaviors			3: 89.8%	1: 12.0%		3: 52.0%
F90 Acts Reduce Distress	1: 4.1%		3: 85.7%	1: 8.0%	2: 8.0%	3: 44.0%
F91 Neither OBS or COMP	No: 100%			No: 96%		
F92 Excessive or Unreasonable	1: 4.1%	2: 2%	3: 87.8%	1: 12.0%		3: 40.0%
F93 Poor Insight	?: 93.9%	1: 4.1%	3: 2.0%	?: 88.0%		1: 4.0%
F94 Symptom Interference (<1 h)	1: 2.0%	2: 2.0%	3: 91.8%	1: 20.0%		3: 20.0%
F95 Axis I Disorder: OBS/ COMP Unrelated	1: 4.1%		3: 83.7%	1: 4.0%	2: 4.0%	3: 8.0%
F96 Not Due to Substance Use or Medical	1: 4.1%		3: 83.7%			3: 16.0%
F97 OCD Criteria A, B, C, D and E are Coded 3	100.0%			4.0%		
F98 Met Criteria for OCD in Past Month	1: 4.1%		3: 69.4%	1: 4.0%		3: 12.0%

“?” = inadequate information, 1 = absent or false, 2 = subthreshhold, 3 = threshold or true; percents may not add up to 100 due to missing data. *p < .05 significance level

When comparing those who were diagnosed correctly versus incorrectly, group differences emerged (see Tables 2 and 3). Fisher’s Exact and Chi-square analyses revealed significant associations between group status (i.e., correct vs. incorrect diagnosis) and several SCID-I screening form and SCID-OCD items. Participants were more likely to receive the correct diagnosis if they answered “yes” on the screening form to the question inquiring about compulsive behaviors (Screen Question #9 - Compulsions), χ^2^ (1, *N* = 74) = 4.90, *p* = .027, OR = 5.88, that was administered at the beginning of the entire interview. Descriptions and examples of obsessions were presented during the interview based on the respondents’ endorsement of obsessive thoughts using the screening form. The prompts used to guide the interviewer inquiries on obsessional thoughts for clarification influenced diagnostic accuracy. Participants were more likely to receive the correct diagnosis if the interviewer followed the line of questioning associated with Screen # 8 prompts (F84a) in the SCID-OCD section, χ^2^ (1, *N* = 74) = 3.89, *p* = .049, OR = 0.22). No other group differences emerged on SCID-I screening form questions or SCID-OCD items.

**Table 3:** Mean scores of African Americans on Clinical Measures who were Correctly and Incorrectly Diagnosed based on the SCID-OCD Section

Clinical Measures	Entire OCD Sample *M* (*SD*)	OCD patients Diagnosed by SCID *M* (*SD*)	OCD patients Misdiagnosed by SCID *M* (*SD*)	*t*
YBOCS Total *	23.47 (14.46)	24.27 (14.14)	21.73 (7.37)	***1.61***
YBOCS Obsessions	10.58 (4.10)	10.94 (3.96)	9.88 (4.35)	1.05
YBOCS Compulsions**	12.18 (3.70)	12.98 (2.89)	10.60 (4.59)	***2.73***
GAF	61.11 (11.48)	61.98 (11.51)	58.95 (11.42)	0.97
CGI (for OCD only)	4.03 (1.07)	4.14 (1.01)	3.83 (1.17)	1.12
BDI-II	28.04 (14.46)	26.20 (14.14)	31.50 (14.71)	1.46
OCI-R Total	42.10 (16.36)	43.49 (16.35)	39.38 (16.38)	1.00
OCI-R Checking	7.71 (3.56)	8.06 (3.31)	7.04 (3.97)	1.17
OCI-R Washing	6.42 (3.89)	6.58 (4.02)	6.12 (3.69)	0.48
OCI-R Ordering*	8.11 (3.77)	8.84 (3.18)	6.63 (4.47)	***1.17***
OCI-R Obsessing	7.15 (3.37)	7.16 (3.41)	7.12 (3.36)	0.05
OCI-R Hoarding	7.46 (3.50)	7.49 (3.47)	7.40 (3.65)	0.10
OCI-R Neutralizing	5.43 (4.10)	5.46 (4.09)	5.38 (4.21)	0.08
OBQ-44 Total	203.72 (51.58)	207.15 (47.98)	196.57 (58.91)	0.81
OBQ-44 Responsibility	70.46 (19.38)	71.90 (19.15)	67.64 (19.90)	0.89
OBQ-44 Perfection	81 (21.04)	84.10 (18.95)	74.92 (23.89)	1.80
OBQ-44 Intolerance	47.54 (16.31)	47.77 (15.11)	47.08 (18.84)	0.17

*N* = 74, *p < .05 significance level, **p < .01 significance level

Independent samples *t*-tests revealed that participants incorrectly diagnosed scored significantly lower on the Y-BOCS [*t*(68) = 1.61, *p* < .05, *d* = 0.39], had less severe compulsive behaviors [*t*(72) = 2.73, *p* < .01, *d* = 0.62], and lower OCI-R Ordering subscale scores [*t*(71) = 1.17, *p* < .05, *d* = 0.57]. There were no other significant group differences on the other continuous measures.

## Discussion

Results indicate that the SCID-OCD lacked the ability to accurately diagnose less severe clinical levels of OCD in African Americans. Those less likely to endorse the presence of symptoms as prompted by the questions of the SCID-OCD section may potentially have limited insight into the severity of their symptoms making them less likely to report when asked in a structured interview format. African Americans seem to have less awareness that OCD represents a potentially serious mental health condition, and about half of those in the current study had not even realized they had a disorder or known how to get treatment for it [44]. Lack of symptom reporting subsequently resulted in the immediate exit out of both the Obsessions and Compulsions section of the SCID-OCD, resulting in an incorrect OCD diagnosis for respondents in this study. The inaccuracy of symptom identification using the SCID to detect OCD in African Americans is problematic, and if other structured interviews perform similarly, this may contribute greatly to African Americans’ underserved status in treatment facilities and underrepresentation population clinical research.

When participants were aware of their compulsive behaviors and reported their symptoms with the screening form, they were more likely to receive an accurate diagnosis than those who did not endorse the presence of rituals. The examples of compulsions/rituals embedded in the questions/prompts may have assisted the respondents in recognizing the ritualistic nature of their behavior. For respondents with less severe symptoms, the examples embedded in the prompts may not have been as useful, resulting in an incorrect OCD diagnosis. Individuals experiencing lower severity of symptoms may require more probing to get information about interference of symptoms, presence of avoidant behaviors, and sense of control over intrusive thoughts and compulsive behaviors. Individuals with types of rituals different than the prompts may also find the examples less helpful, making it less likely for them to report their symptoms using this interviewing format. For example, washing, checking, and counting rituals are more easily recognized as overt compulsive behaviors in OCD [45, 46]; therefore, inquiring about these types of rituals could result in greater endorsement. That being said, African Americans are more likely to have contamination concerns compared with European Americans, so cultural differences in symptom presentation are unlikely to be the sole cause of the problem [47]. The screening questions were removed from the SCID-5, which could improve identification of OCD. Finding ways to assist the client in gaining awareness of the pathological nature of ritualistic behaviors might also result in easier identification of OCD symptoms.

In the SCID-OCD Obsessions section, there are two examples (“hurting someone” and “germs or dirt”) that facilitated the correct diagnosis of OCD in African Americans. However, people with OCD may become preoccupied about many different things, such as worries about animals or fears of being misunderstood [47]. If respondents do not have obsessive thoughts about hurting someone or dirt, respondents would not meet criteria for obsessions according to the SCID-OCD section resulting in an immediate exit of the section. Unfortunately, this does not allow for additional probing of symptoms as found in other interviews (e.g., Anxiety Disorders Interview Schedule-OCD Section [48, 49]). Additionally, less obvious rituals like mental checking may be viewed as natural, reasonable, or appropriate. Individuals performing these rituals are less likely to acknowledge the interference of these “natural” rituals, which decreases respondents’ ability to recognize their compulsions as maladaptive or part of a disorder during an interview [50]. In this case, question formatting may also influence whether people are able to recognize their symptoms, impacting the likelihood of their reporting. These individuals may require more probing, which the SCID-OCD’s question formatting limited, resulting in a higher likelihood of a missed diagnosis. The SCID-5 distinguishes between images, urges, and thoughts, allowing for further probing into different types of obsessions that could increase improve symptom endorsement.

Participants reporting ordering compulsions in this study had a higher likelihood of having their symptoms accurately assessed by the SCID-OCD section. Neither prompt leading into the assessment of compulsions mentions ordering rituals as an example, yet there were significant group differences on the OCI-R Ordering Subscale between those accurately and inaccurately diagnosed. Our findings may be a reflection of our sample, given that over half reported higher levels of ordering compulsions on the OCI-R, endorsing three or higher on all three subscale questions (53% with score of 9–12 on OCI-R Ordering Subscale).

These findings could also indicate a need for the SCID-OCD to include more information about different types of symptoms during the interview, similar to the Anxiety Disorder Interview Schedule-Obsessive-Compulsive Disorder (ADIS-OCD) section. The SCID-OCD could be improved to detect OCD among African Americans by including examples of symptoms that are more common among this group. For example, the fear of being misunderstood seems to be much more common among African Americans with OCD, experienced by almost half of those with the disorder [47], so this could be added as one of several additional prompts. Additionally, it is vitally important that clinicians understand the cultural differences across the ethnic groups they serve, as OCD can take on varied presentations that may be culture-specific [51]. The SCID-5 OCD section has undergone changes simplifying the assessment of diagnostic criteria, but concerns regarding culturally-specific presentations may still exist. As such, important recommendations include formal training in cultural competence, development of multi-cultural awareness, and having an ethnically and racially diverse staff [52].

### Limitations

Limitations of the present study include the cross-sectional and correlational design, which precludes us from inferring causality. Future research with this instrument and in this population would benefit from examining all types of clinical utility (e.g., true negatives), perhaps by incorporating control groups. Comparing the utility of this instrument to the newly released version would further elucidate the effectiveness of the SCID as a diagnostic tool for OCD. Additionally, since we only examined one ethnoracial group, we do not know if the findings are specific to African Americans or other groups as well. Future research should examine the relationship of ethnicity, symptom dimensions, and utility of each section of the SCID in assessing Axis I Disorders in African Americans and other ethnoracial groups.

We did not determine if experience with the SCID-OCD was a factor in missed diagnoses. All evaluators were well trained in the use of the SCID-OCD and in the assessment of OCD, but most were not expert OCD clinicians. Previous literature indicates that expert knowledge of OCD results in better outcomes with the measure, but instruments such as the SCID-OCD should be effective when used by knowledgeable clinicians who are not necessarily experts in every disorder. Another limitation of the interviews was that order of administration was not counterbalanced, so results from one interview (e.g., SCID-I) could have contaminated results from another (e.g., Y-BOCS), since the SCID-I was administered before the Y-BOCS in all cases.

The sample size yielded enough statistical power to identify medium and large effects, but the study was underpowered to detect small effects. Concerns for statistical power also precluded adjusting alpha for multiple pairwise comparisons. Thus, future research should incorporate larger samples to enhance statistical power and allow for adjusting for Type I error rate inflation. Additionally, future research on ethnic and racial differences on the SCID-OCD would be strengthened by adopting a mixed-methods approach, including the use of cognitive interviewing techniques, as a way of establishing qualitative patterns of language and interpersonal style among participants who are completing a semi-structured diagnostic interview.

## Conclusion

The accurate diagnosis of OCD remains a difficult task that requires a specialized set of skills and familiarity with the disorder. Given that African Americans are less likely to recognize their symptoms as being indicative of OCD [44], clinicians having familiarity with OCD symptoms and being well-trained in its assessment would no doubt improve the likelihood of correctly diagnosing the presence of the disorder. However, expertise with OCD may not be sufficient to render an accurate diagnosis using the SCID-OCD alone. More symptom specific assessment tools, such as the Y-BOCS, Dimensional Obsessive-Compulsive Scale (DOCS) [53], and OCI-R should be administered to help ensure an accurate understanding of symptoms. Not all assessment tools are valid for use with all groups, and interviewers need to be aware of such limitations when assessing OCD in African American subjects. Future research should continue to validate clinical measures and related assessment tools in African Americans to determine their accuracy in assessing and diagnosing OCD and related symptoms. Specifically, more research should be done to investigate how well structured interviews capture OCD in various ethnic groups.
